# Sociodemographic predictors of knowledge, mosquito bite patterns and protective behaviors concerning vector borne disease: The case of dengue fever in Chinese subtropical city, Hong Kong

**DOI:** 10.1371/journal.pntd.0008993

**Published:** 2021-01-19

**Authors:** Emily Ying Yang Chan, Eugene Siu Kai Lo, Zhe Huang, Holly Ching Yu Lam, May Pui-shan Yeung, Kin-on Kwok, Kevin Kei Ching Hung, Shelly Lap-ah Tse

**Affiliations:** 1 Collaborating Centre for Oxford University and CUHK for Disaster and Medical Humanitarian Response (CCOUC), JC (Jockey Club) School of Public Health and Primary Care, Faculty of Medicine, Chinese University of Hong Kong, Hong Kong Special Administrative Region, China; 2 Nuffield Department of Medicine, University of Oxford, Oxford, United Kingdom; 3 JC (Jockey Club) School of Public Health and Primary Care, Faculty of Medicine, Chinese University of Hong Kong, Hong Kong Special Administrative Region, China; 4 Stanley Ho Centre for Emerging Infectious Diseases, The Chinese University of Hong Kong, Hong Kong Special Administrative Region, China; 5 Shenzhen Research Institute of the Chinese University of Hong Kong, Shenzhen, China; 6 Accident and Emergency Medicine Academic Unit, Prince of Wales Hospital, Hong Kong Special Administrative Region, China; Oregon State University College of Veterinary Medicine, UNITED STATES

## Abstract

Geographic pattern of dengue fever is changing due to the global environmental and climate changes in the 21^st^ century. Evidence of community’s knowledge, mosquito bite patterns and protective behavior practices in non-endemic regions is limited. This study examined the knowledge of dengue, mosquito bite patterns, protective behavior practices and their associated factors in Hong Kong, a non-endemic subtropical city. A population-based random telephone survey (n = 590) was conducted three weeks after the government announcement of a local dengue outbreak in August 2018. Sociodemographic status, awareness, knowledge, protective measures, bite patterns of mosquito were collected. Results indicated high level of community awareness of the local outbreak (95.2%), symptom identification (84.0%) and adoption of at least one mosquito protective measures (nearly 80%). About 40% of respondents reported that they were bitten by mosquitoes during the study period, a high mosquito season in Hong Kong. Mosquito bites were prevalent near grassy area (63.4%), at home (42.6%) and at public transportation waiting spots (39.6%). Younger people (< 25 years old), female, those who lived on lower floors (≤the 6^th^) and near grassy area were at higher risk of mosquito bites at home. Respondents perceived higher threat of dengue to society were more likely to practice mosquito prevention. While residential factors affected their indoor prevention, other socio-demographic factors affected the outdoor prevention. Practicing prevention behaviors were associated with self-reported mosquito bite at home. Furthermore, the general prevention uptake rate unchanged after the announcement of local dengue outbreak. Although the uptake rate of protective measures during August was high, 40% participants reported they were bitten. Also public locations are more common area for bites, which suggested stronger mosquito prevention and control on public environments and more personal protective behaviors should be advocated.

## Author summary

Dengue is a mosquito-borne viral disease that has rapidly spread globally in recent years. It is transmitted by female mosquitoes by *Aedes aegypti* and *Aedes albopictus*. The best prevention method is to control mosquito population and avoid being bitten. This study is a telephone survey which examined the knowledge level towards dengue, patterns of mosquito bite and protective behavior among Hong Kong population, and is the first study examining subtropical urban knowledge, mosquito bite patterns and protective measures adoptions against mosquito. In August 2018, 590 Hong Kong residents were interviewed. Results indicated that about 4 out of 5 respondents could identify 1 symptom of dengue and adopt at least 1 mosquito prevention behavior. About 40% respondents claimed they were bitten by mosquitoes in August. The most prevalent places were near grassy area (63.4%), home (42.6%) and transportation waiting spots (39.6%). Younger age, female, living on lower level (6^th^ floor or lower), and near grassy area were associated with self-reported mosquito bite at home. Respondents believing that dengue has a large impact to the society were more likely to adopt protective measures. While responders’ residential factors affected their adoption of indoor protective measures, other socio-demographic factors affected their outdoor adoption of protection.

## Introduction

### The global prevalence and trend of dengue fever

Dengue fever is a mosquito-borne infectious disease with more than 100 tropical and subtropical countries globally reported to be endemic[1] and it was estimated that more than half of the total population were at risk in the world[2]. According to the World Health Organization, the estimated annual fatality rate [1] was 2.5%, among individuals with severe dengue, with complications like hemorrhagic fever and fluid accumulation. Dengue infection is prevalent in Southeast Asia[3] because of the presence of its primary vector *Aedes aegypti* mosquitos. In regions without this vector, including European and North American areas, imported cases and the potential outbreak are possible due to the existence of a second mosquito vector, *Aedes albopictus*. Dengue infection was commonly reported among children and teenagers[4] but the prevalence among older age groups was also found increasing in recent decades[5].

Associated with the increasing average temperature and alterations of precipitation caused by climate change, dengue outbreak risks have been reported or projected to increase in non-endemic regions[6,7]. For instance, an unexpected dengue outbreak was reported in 2014 in Japan where there had no reported dengue cases before[8]. Thus, non-endemic communities without preparedness may be under increasing risks of dengue outbreak in response to the climate change catastrophe.

### The literature on dengue fever prevention at household and personal level

In terms of primary prevention of dengue fever, protective measures against mosquitoes at household- and individual-level were the focus of most published studies. Effectiveness of the use of mosquito net in controlling dengue was widely studied in endemic regions such as South Africa and Southeast Asia[9–11]. Risk perception[12] and socio-demographic status[13] were reported to be the associated factors of the adoption of protective measures. Community knowledge level was found to be related to active disease detection and treatment seeking behavior. Nevertheless, research articles about the knowledge, attitude as well as management of dengue fever mainly focused on at-risk countries (mostly countries in the tropical region) such as Nepal[14], Indonesia[15], India[16] and Singapore[17], limited research work had been done within non-endemic regions despite of their increasing risks.

### Mosquito surveillance and prevention and the 2018 dengue outbreak in Hong Kong

Hong Kong is a subtropical city with the existence of the secondary vector, *Aedes albopictus*. The government has implemented strategic plans such as vector control, effective epidemiological surveillance (case diagnosis and referral), entomological surveillance (ovitrap index), emergency preparedness and capacity building[18], to prevent mosquito-related public health problems and diseases outbreaks since 2005. Ovitrap Index is a simple early warning system for *Aedes albopictus* breeding and the risk of dengue in Hong Kong. As of August 2018, there were 57 ovitrap monitoring locations in Hong Kong to monitor the territory-wide prevalence of *Aedes albopictus*[19]. Health promotion campaign was also provided to the public to raise the awareness of applying protective measures including removing stagnant water (the main focus of the campaign), wearing light colored long sleeve clothes and using mosquito repellent[20].

On 14 August 2018, a first local dengue outbreak [21] with 153 dengue fever confirmed cases from January 2018 to November 2018 occurred in Hong Kong. With 29 (18.95%) of them were identified as local cases[22], this outbreak was the largest-ever on record in terms of the number of local cases [23].

### The vulnerability of Hong Kong, the gaps to be filled and the objectives of this study

Although the majority of the dengue fever infections in Hong Kong were imported cases[24], it was believed that the *Aedes albopictus* adult mosquito production would increase[25] due to the rising temperature caused by global warming and continuously urbanization in Hong Kong[26]. The 2018 outbreak indicated an urgent need to strengthen the prevention strategies. Scientific evidence, such as pattern of mosquito bites, community’s knowledge, risk perception and current protective practices about mosquitoes, could assist in effective policy development to protect the population from dengue fever. This study investigated 1) awareness of the 2018 outbreak and dengue related knowledge, 2) the pattern of mosquito bites, 3) the prevalence of the use of mosquito protective measures, 4) the associated factors of the adoption of mosquito protective measures and 5) the possible relationship between the proxy measure, ovitrap index, and mosquito protective behaviors towards self-reported mosquito bites.

## Methods

### Ethics statement

This study was approved by the Survey and Behavioural Research Ethics Board, The Chinese University of Hong Kong. Verbal consent was obtained from the participants and ethics approval and the ethical approval was obtained from the Survey and Behavioral Research Ethics committee of the Chinese University of Hong Kong.

### Hypotheses

Regarding this study, there were three hypotheses:

1. Sociodemographic factors (e.g. the age and education, and residential characteristics), ovitrap data, adoption of mosquito protective measures were associated with mosquito bite at home.
2. Sociodemographic factors were associated with the adoption of mosquito protective measures.
3. The adoption of mosquito preventive measures after the local outbreak was higher than before.

### Study design and study population

A cross-sectional population-based telephone survey was conducted in September 2018, 3 weeks (6 Sep 2018 to 15 Sep 2018) after the government declaration of the dengue fever outbreak on 14 August 2018[27]. Study respondents were randomly chosen from the landline telephone list covering 89.36% of households in Hong Kong[28]. At most five times of calling were attempted on each number. Individuals responded with non-residential landline were not included. To avoid selection bias of the phone respondents, the person with birthday closest to the calling date was invited to the survey [29]. The recruited population was compared with the Hong Kong population census in 2016 with respect to their age group, gender and residential districts. Hong Kong residents aged 18 years or above were recruited, including those who hold valid working or study visa. Individuals who cannot speak Cantonese and were not in Hong Kong from April to August 2018 were excluded.

### Measurement and mosquito-related behaviors and other mosquito-related information

In the survey, respondents were asked about their awareness of the 2018 local dengue outbreak and their understanding of dengue fever, including the transmission route, the perception toward dengue fever, mosquito protective measures, and pattern of mosquito bites within August 2018 (including time and locations). Individual and household level protective measures adopted against mosquitos before (14 Jul– 13 Aug) and after the government declaration of dengue outbreak (14 Aug– 14 Sep) were collected. Indoor measures included having mosquito-eating plants, using anti-mosquito detergent, incense, insecticide, electronic repellent, mosquito net (on door, on window or for bed), removing stagnant water, while outdoor measures included using chemical repellent, stickers, bracelet, and wearing light colored long sleeves clothes. Protective measure before the outbreak announcement was used in this study these represent people’s usual behavior. In the subsequent analysis, the measures were analyzed in 4 groups: “adopt any indoor measures”, “adopt any outdoor measures”, “adopted any mosquito measures”, and “adopted 3 or more mosquito measures”. Finally, reasons for not doing any protective measures were asked. For ovitrap data collection, the regional ovitrap index data from May to August 2018 were obtained from the official webpage of the Food and Environmental Hygiene Department[30]. Each respondent was matched with the ovitrap index data geographically according to their reported residential districts. For those residential districts covering more than one ovitrap regions, the involved ovitrap indices were averaged.

### Statistical analysis

Descriptive statistics were used to report the socio-demographic characteristics of respondents, awareness of the local dengue outbreak, the relevant knowledge, risk perception toward dengue fever, mosquito bites patterns and the adoption of protective measures before and after the announcement of dengue outbreak. McNemar’s test was used to compare the difference of the mosquito measures between before and after local outbreak in Hong Kong. Bivariate analyses were used for identifying the associated factors of home mosquito bites and the uptake of protective behaviors. Explanatory variables included sociodemographic factors, housing factor, perception on dengue risk to society, perceived risk of getting dengue fever in Hong Kong and the perceived usefulness of individual/household prevention. Explanatory variables were then put into multivariable logistic regression if they had P-value<0.1. Statistical significance was set 0.05. In addition, multivariable logistic regression analysis, adjusting for the significant predictors to mosquito bites in the previous regression results, was used for identifying the relationship of the protective measures and the self-reported mosquitos bite at home. The uptake of the protective behaviors before the outbreak were used in the logistic regression model analysis, as they would represent the usual practices of Hong Kong residents. We also performed weighted analyses based on the categories of age group and education attainment distribution in the population and our sample, since their proportions between them were relatively large as showed in Table 1. Age group had four categories and education attainment had three, which led to 12 different groups of age group and education combination. According to the population percentage of each combination of age group and education, a weight was calculated for each group in the sample. The results of weighted analyses where both age group and education level were weighted were presented in S1–S6 Tables, which were comparable with the results from the unweighted analysis. All statistical analyses were conducted using IBM SPSS 21 for Windows.

**Table 1:** The respondent population details and the comparison with Census population data.

	2016 population census	Study sample (N = 590)
Age		
18–24	9.50%	70 (11.86%)
25–44	35.26%	150 (25.42%)
45–64	36.84%	231 (39.15%)
65 or older	18.40%	139 (23.56%)
Gender		
Male	45.10%	249 (42.20%)
Female	54.90%	341 (57.80%)
Marital status		
Non-married	39.93%	240 (41.59%)
Married	60.07%	337 (57.12%)
Residential district^a^^,^^b^		
Hong Kong Island	17.22%	107 (18.14%)
Kowloon	30.55%	181 (30.68%)
New Territory	52.23%	302 (51.19%)
Education attainment^a^		
Primary or below	25.72%	87 (14.87%)
Secondary	43.67%	255 (43.59%)
Tertiary	30.60%	243 (41.54%)

^a^The Hong Kong population Census data additionally included age 15 to 17 years old.

^b^Marine population was excluded.

## Results

**Fig 1:**
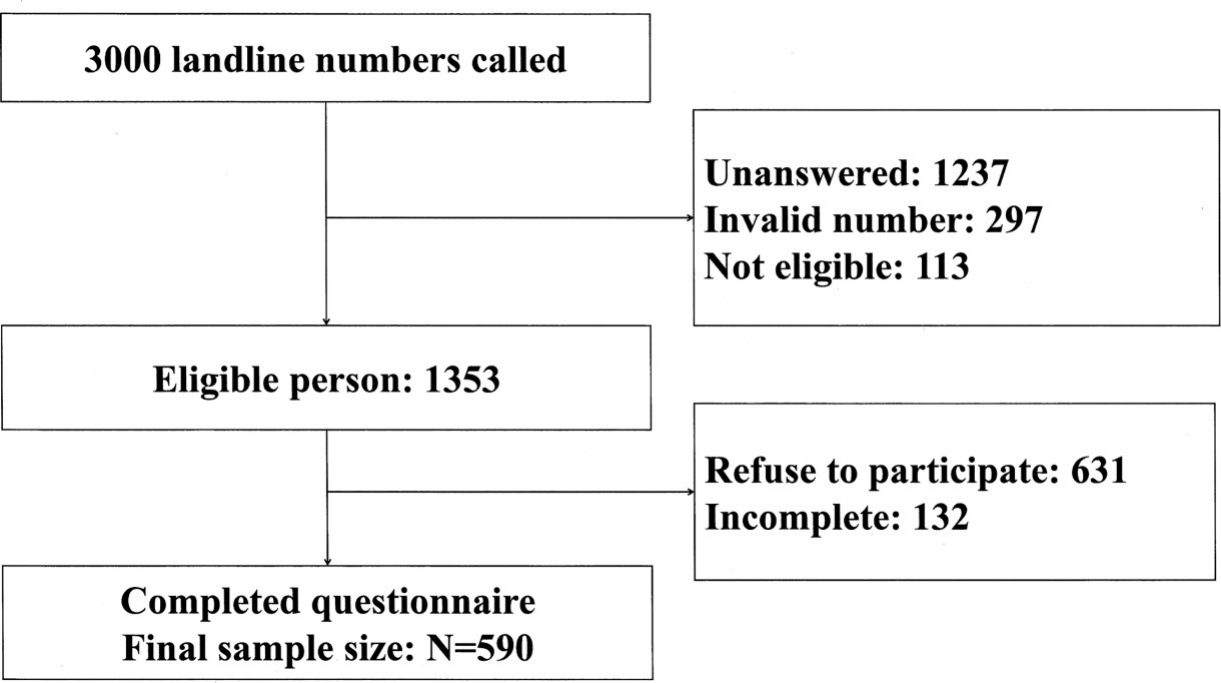
The recruitment details in the telephone survey.

In total, 1353 eligible persons were reached and 590 respondents (response rate: 43.6%) were successfully recruited (Fig 1).

### Socio-demographic characteristics, awareness of the 2018 outbreak, knowledge of dengue fever and risk perception

The study sample was comparable with the population data in Hong Kong Census 2016[31] with respect to their gender, marital status and living districts (Table 1).

### Knowledge related to dengue fever

**Fig 2:**
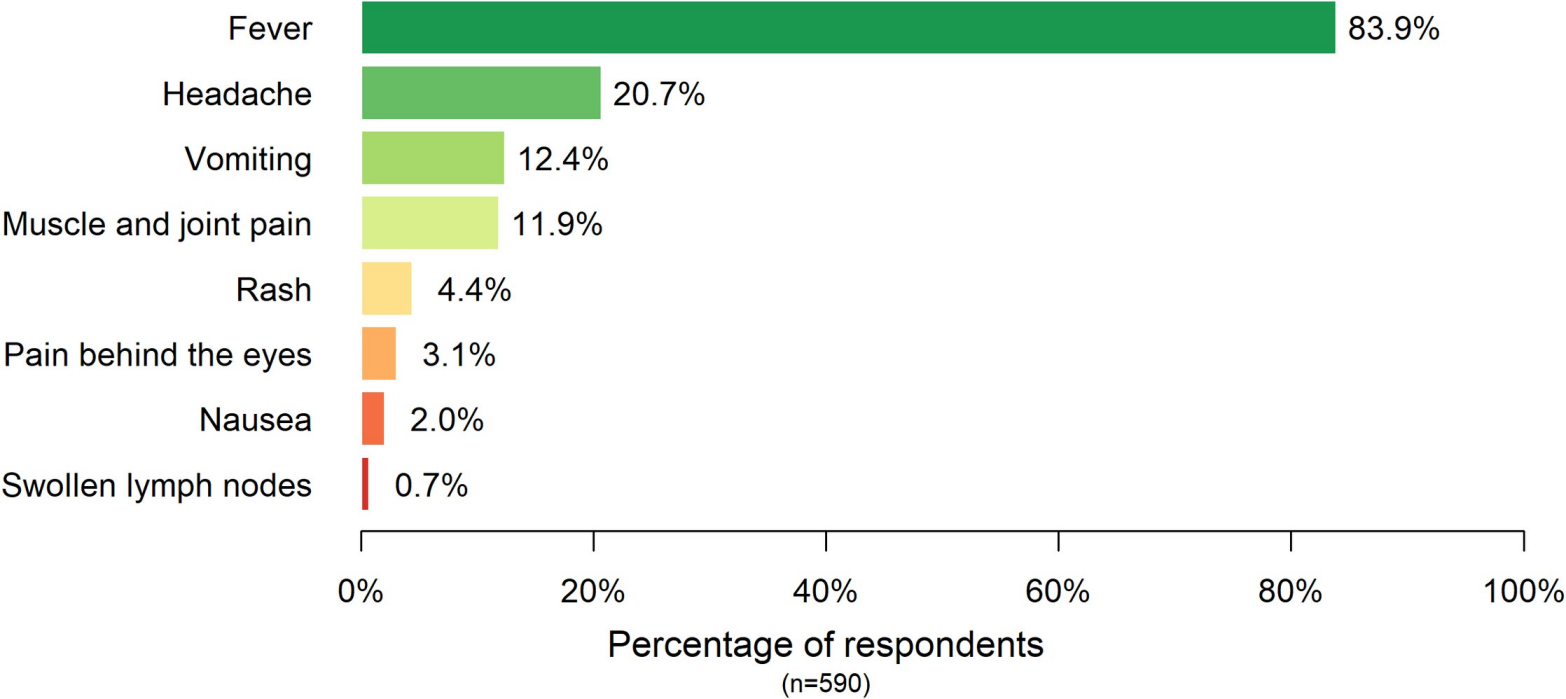
Symptoms of dengue fever recognized by respondents in September 2018. Note: Respondents can choose more than 1 answer.

In this study, 562 respondents out of 590 (95.3%) reported that they noticed the announcement of local dengue fever outbreak by Hong Kong government in August 2018. For knowledge related to dengue fever, 544 out of 590 (92.2%) understood that dengue fever could be transmitted by mosquitoes and 473 of them (87.6%) correctly indicated that *Aedes* mosquitoes are the vector of dengue fever. For symptoms of dengue fever (Fig 2 and S1 Table), most of the respondents (84.0%) knew that fever was one of the symptoms, but comparatively fewer of them could recognize other related symptoms, such as headache (20.7%) and vomiting (12.4%).

### Risk perception of dengue fever infection

Around 72.1% respondents believed that the risk for getting dengue fever in Hong Kong was either “low” or “very low”, while about half of the respondents (51.6%) believed the risk for getting dengue fever in foreign regions was “low” or “very low”. Around half (51.4%) of the respondents reported that they would completely avoid going to locations with confirmed dengue fever cases such as Wong Tai Sin and Cheung Chau in Hong Kong during the 2018 outbreak.

### Patterns of mosquito bites

Out of the 590 respondents, 235 (39.8%) reported that they were bitten by mosquitoes in August 2018. Among those reported being bitten, 26 (11.1%) indicated they have been bitten daily. In addition, 58 out of 590 respondents (9.8%) reported that mosquito bites were affecting their daily lives.

**Fig 3:**
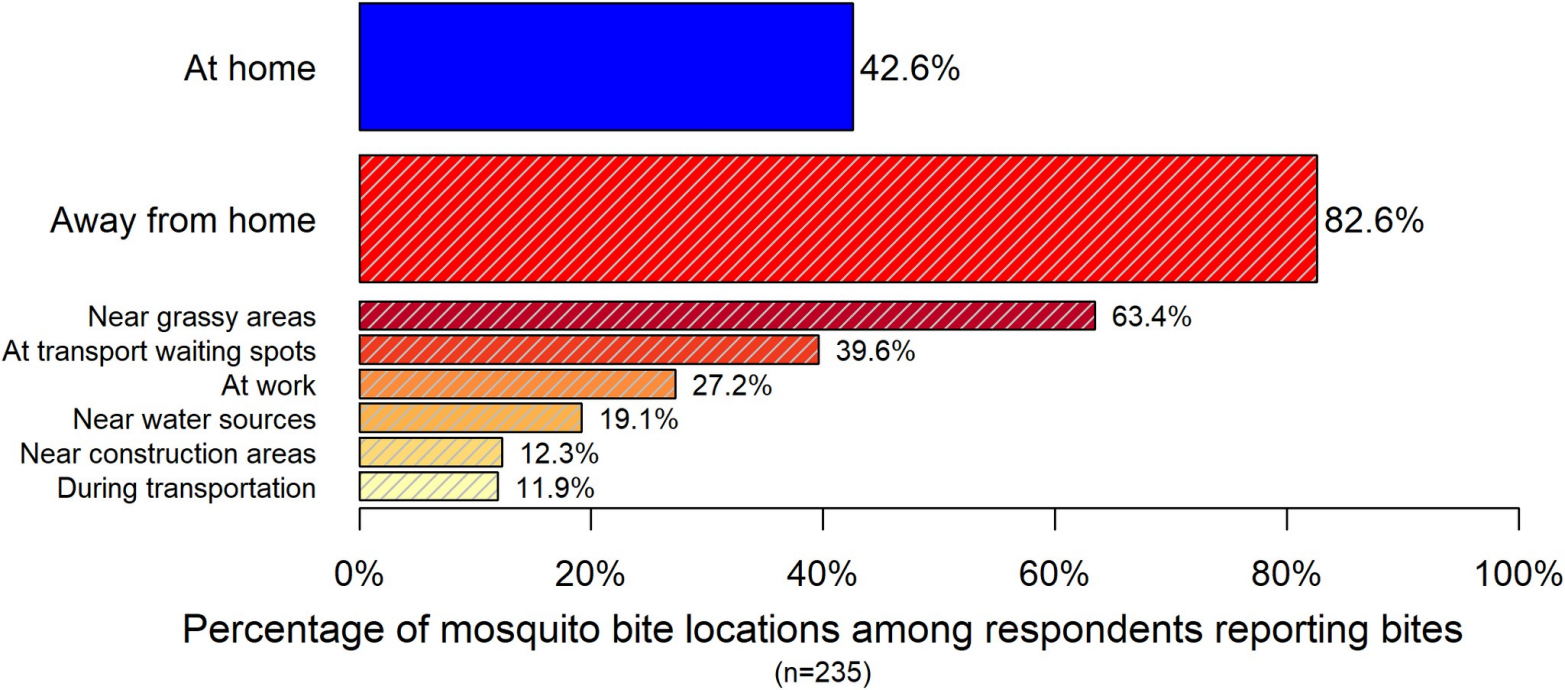
The locations of mosquito bites in August 2018. Note: reporting bites near grassy areas, at transport waiting spots, at work, near water sources, near construction areas and during transportation were merged as away from home. Respondents can choose more than 1 answer.

Among the respondents reported mosquito bites (Fig 3 and S1 Table), 42.6% reported they were bitten at home, and 82.6% reported bites away from home. Bites were most commonly reported at transportation waiting spots (39.6%) and near grassy areas (63.4%).

### Associated factors of mosquito bites at home

Bivariate analysis and multivariable logistic regression were performed to investigate the associated factors of self-reported mosquito bites at home (Tables 2 and S2). Respondents of younger age, female gender, living 6 floor or lower and near grassy area like parks, garden, hills were shown to be significantly associated with more mosquito bites at home.

**Table 2:** Associated factors of mosquito bites at home.

Factors	No mosquito bites (n = 472)	Have mosquito bites (n = 100)	p-value^#^	AOR (95% CI)	p-value^$^
Age			<0.001*		
18–24	48 (10.2%)	20 (20.0%)		1	
25–44	109 (23.1%)	37 (37.0%)		0.707 (0.360–1.390)	0.315
45–64	189 (40.0%)	32 (32.0%)		0.380 (0.181–0.801)	0.011*
65 or older	126 (26.7%)	11 (1.0%)		0.251 (0.096–0.655)	0.005*
Gender			0.024*		
Male	209 (44.3%)	32 (32.0%)		1	
Female	263 (55.7%)	68 (68.0%)		1.958 (1.186–3.230)	0.009*
Residential district			0.420		
Hong Kong Island	90 (19.1%)	14 (14.0%)			
Kowloon	139 (29.4%)	34 (34.0%)			
New Territories	243 (51.5%)	52 (52.0%)			
Housing			0.971		
Public housing	167 (35.5%)	36 (36.4%)			
Support housing	69 (14.6%)	16 (16.2%)			
Private housing	230 (48.8%)	46 (46.5%)			
Temporary housing	5 (1.1%)	1 (1.0%)			
Floor Level			<0.001*		
<6	90 (19.1%)	37 (37.4%)		1	
6–25	284 (60.4%)	53 (53.5%)		0.450 (0.270–0.750)	0.002*
>25	96 (20.4%)	9 (9.1%)		0.222 (0.099–0.498)	<0.001*
Live near water source			0.252		
No	268 (57.3%)	51 (51.0%)			
Yes	200 (42.7%)	49 (49.0%)			
Live near bushy and grassy area			0.029*		
No	46 (9.7%)	3 (3.0%)		1	
Yes	426 (90.3%)	97 (97.0%)		3.411 (1.007–11.550)	0.049*
Live near construction site			0.183		
No	310 (66.%)	58 (59.2%)			
Yes	158 (33.8%)	40 (40.8%)			
CSSA			0.179		
No	444 (94.9%)	97 (98.0%)			
Yes	24 (5.1%)	2 (2.0%)			
Education			0.028*		
primary and below	76 (16.3%)	8 (8.0%)		1	
secondary	206 (44.1%)	40 (40.0%)		1.396 (0.583–3.342)	0.454
post-secondary	185 (39.6%)	52 (52.0%)		1.351 (0.518–3.520)	0.538
Chronic disease			0.024*		
No	348 (75.2%)	84 (85.7%)		1	
Yes	115 (24.8%)	14 (14.3%)		0.779 (0.389–1.558)	0.480
Regional ovitrap index	6.47 (4.97–11.05)	6.17 (4.97–11.05)	0.719		

*p<0.05.

# Chi-square or Mann-Whitney U test.

$ Multivariable logistic regression.

### Patterns and the associated factors of protective measures adoption against mosquito bites

#### The prevalence of the use of mosquito protective measures

In our sample, 363 (61.5%) respondents believed that individual or household protection could prevent dengue infection. About 77.8% (459 out of 590) and 77.3% (456 out of 590) respondents reported that they had performed one or more protective measures against mosquito bites before and after the local case announcement (Tables 3 and S3). Clearing stagnant water (51.0%), using mosquito repellent (39.8%) and wearing light colored long clothing (28.1%) are the most common adopted protective measures. McNemar’s tests found that there was an increase uptake for removing stagnant water, and decrease uptakes in applying electronic repellent, general outdoor mosquito measures and the use of mosquito bracelet.

**Table 3:** Uptake rate of protective measures (N = 590).

Mosquito protective measures	Before the local outbreak		After the local outbreak		McNemar’s test
	N	%	N	%	p
**Indoor mosquito measures**	394	66.8	400	67.8	0.345
Removing stagnant water	300	50.8	327	55.5	<0.001
Electronic repellent	117	19.8	89	15.1	<0.001
Insecticide	111	18.8	105	17.8	0.238
Mosquito net on door and window	75	12.7	73	12.4	0.625
Mosquito incense	51	8.6	48	8.1	0.549
Mosquito plant	16	2.7	14	2.4	0.500
Mosquito net during sleep	15	2.5	15	2.5	1.000
Detergent cleaning	14	2.4	15	2.5	1.000
**Outdoor mosquito measures**	314	53.2	300	50.8	0.049
Mosquito chemical repellent	234	39.7	225	38.3	0.243
Light colored long clothing	166	28.1	173	29.3	0.230
Mosquito sticker	110	18.6	103	17.5	0.265
Mosquito bracelet	38	6.4	25	4.2	0.001

#### Reasons for not adopting anti-mosquito measures

Around one-fifth (22.2%) of respondents reported they had not done any protective measures. Among them, 113 (86.3%) had provided reasons for not applying any measures. The main reason was “it is not necessary” (61.1%). Only a few of them reported “I have not thought of doing it” (3.5%), “I don’t know how to do” (0.9%), “It is no use” (0.9%) and others (33.6%).

#### Associated factors of mosquito protective measures (indoor, outdoor, adopt at least 1 protective measure, and adopt 3 or more protective measures)

Bivariate analysis and multivariable logistic regression were performed to investigate the associated factors of the uptake of indoor and outdoor mosquito protective measures, the adoption of at least 1 protective measure, and adoption of 3 or more protective measures” (Tables 4 and 5 and S4 and S5).

**Table 4:** Associated factors of the use of indoor and outdoor protective measures against mosquito bites.

	Adopt indoor measure		Adopt outdoor measure	
	AOR (95% CI)	p	AOR (95% CI)	p
Age				
18–24	1		1	
25–44	0.933 (0.490–1.776)	0.832	1.324 (0.696–2.521)	0.392
45–64	1.244 (0.668–2.316)	0.491	0.732 (0.385–1.393)	0.342
65 or older	1.330 (0.685–2.581)	0.399	0.486 (0.239–0.988)	0.046*
Gender				
Male	1		1	
Female	1.249 (0.851–1.833)	0.257	2.226 (1.501–3.302)	<0.001*
Residential district				
Hong Kong Island	1		1	
Kowloon	1.453 (0.850–2.481)	0.172	1.226 (0.715–2.104)	0.459
New Territories	1.688 (1.028–2.773)	0.039*	1.622 (0.987–2.663)	0.056
Floor level				
<6	1			
6–25	0.493 (0.297–0.816)	0.006*		
>25	0.518 (0.280–0.957)	0.036*		
Live near water source				
No			1	
Yes			1.190 (0.801–1.768)	0.390
Live near bushy, grass area				
No	1		1	
Yes	2.194 (1.162–4.142)	0.015*	1.433 (0.725–2.830)	0.300
Live near construction site				
No	1		1	
Yes	1.628 (1.089–2.434)	0.018*	1.440 (0.974–2.128)	0.068
Education				
Primary and below			1	
Secondary			2.344 (1.281–4.288)	0.006*
Post-secondary			2.971 (1.495–5.905)	0.002*
Perceived mosquito bites affecting their daily life (Mosquito Annoyance)				
No	1		1	
Yes	1.977 (0.887–4.407)	0.096	1.182 (0.586–2.382)	0.640
Dengue fever could be avoided through individual/household prevention				
Disagree/Neutral	1		1	
Agree	1.531 (1.040–2.255)	0.031*	1.292 (0.882–1.893)	0.188
The impact of dengue toward the whole society				
Low	1		1	
Medium	1.653 (1.083–2.522)	0.020*	1.930 (1.265–2.946)	0.002*
High	1.814 (1.094–3.008)	0.021*	1.811 (1.104–2.972)	0.019*
Risk for getting dengue fever in Hong Kong				
Very low/low	1		1	
Medium/ very high	0.973 (0.617–1.534)	0.907	1.372 (0.871–2.161)	0.173

*p<0.05.

**Table 5:** Associated factors of the use of protective measures against mosquito bites.

	Adopt at least 1 protective measure		Adopt 3 or more protective measures	
	AOR (95% CI)	p	AOR (95% CI)	p
Age				
18–24	1		1	
25–44	1.228 (0.551–2.735)	0.616	0.778 (0.412–1.470)	0.440
45–64	0.776 (0.371–1.621)	0.499	0.481 (0.245–0.942)	0.033*
65 or older	0.784 (0.361–1.703)	0.539	0.411 (0.190–0.890)	0.024*
Gender				
Male	1		1	
Female	1.490 (0.958–2.319)	0.077	1.845 (1.209–2.817)	0.005*
Residential district				
Hong Kong Island			1	
Kowloon			0.850 (0.456–1.583)	0.608
New Territories			1.958 (1.125–3.408)	0.017*
Floor level				
<6	1		1	
6–25	0.488 (0.265–0.897)	0.021*	0.370 (0.228–0.602)	<0.001*
>25	0.555 (0.266–1.160)	0.117	0.371 (0.201–0.683)	0.001*
Live near water source				
No	1		1	
Yes	1.081 (0.683–1.712)	0.740	1.216 (0.801–1.847)	0.358
Live near bushy, grass area				
No	1		1	
Yes	2.783 (1.399–5.537)	0.004*	1.384 (0.624–3.070)	0.424
Live near construction site				
No	1		1	
Yes	1.965 (1.210–3.190)	0.006*	1.783 (1.179–2.697)	0.006*
Education				
Primary and below			1	
Secondary			3.690 (1.722–7.909)	0.001*
Post-secondary			2.719 (1.179–6.269)	0.019*
Perceived mosquito bites affecting their daily life (Mosquito Annoyance)				
No	1		1	
Yes	2.132(0.702–6.474)	0.182	1.940 (0.972–3.869)	0.060
Dengue fever could be prevented through individual/household protection				
Disagree/Neutral	1		1	
Agree	1.064 (0.682–1.658)	0.786	2.161 (1.398–3.342)	0.001*
The impact of dengue toward the whole society				
Low	1		1	
Medium	2.139 (1.304–3.506)	0.003*	1.254 (0.794–1.980)	0.332
High	1.958 (1.088–3.522)	0.025*	1.694 (1.001–2.867)	0.050*
Risk for getting dengue fever in Hong Kong				
Very low/low	1		1	
Medium/ very high	1.085 (0.629–1.873)	0.769	1.153 (0.720–1.847)	0.554

*p<0.05.

Living in New Territories, living on lower level (6 floors or lower), living near bushy, grass area and construction site, believing individual/household protection could prevent dengue fever, and perceiving dengue has higher impact toward whole society were associated with adopting “indoor measures”. As for the “outdoor measures”, younger age (aged 18–24 comparing to 65 aged or older), female gender, having higher education level, perceiving higher dengue impact toward whole society were found to be the significant positive factors of adoption.

Respondents who were living at lower floors, near bushy and grassy area as well as construction sites, believing that individual protection could prevent dengue, and perceiving the higher impact of dengue fever toward society were more likely to adopt at least 1 protective measure. For those who had adopted 3 or more listed measures, younger age, female gender, living in New Territories, higher education level, resided on floor level below 6, living near construction site, believing dengue could be avoided by individual protection and perceiving higher impact of dengue fever toward society were the significant associated factors.

### Relationships between ovitrap index and protective measures adoption toward self-reported mosquito bite at home

**Fig 4:**
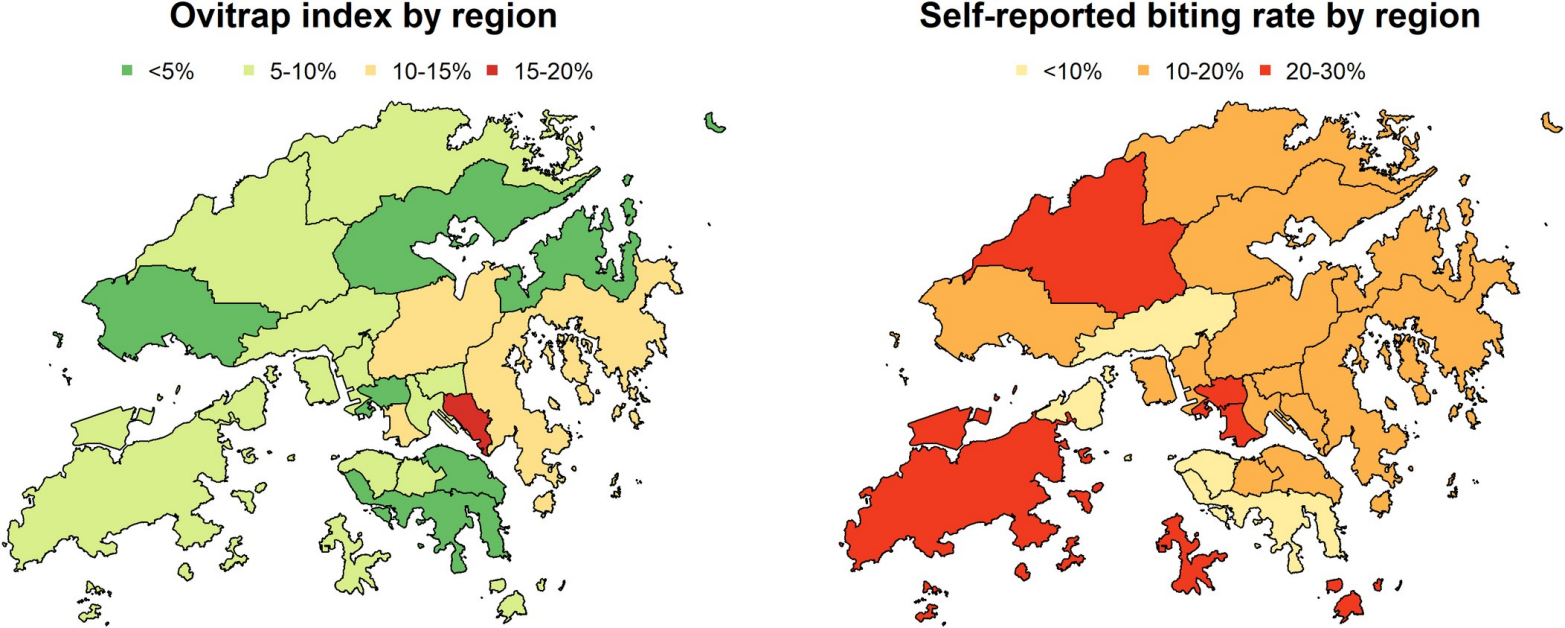
Maps of ovitrap index and self-reported biting rate in August 2018. Source: Based on the data from Hong Kong SAR Government Planning Department, 2016 Tertiary Planning Unit and Street Block/Village Cluster (TPU&SB/VC) Boundaries.

Associations between regional ovitrap index and self-reported mosquito bite at home were not statistically significant as shown in Table 2. Fig 4 described the ovitrap index and self-reported biting rate by region in August 2018 (S1 Data). Multivariable logistic regressions were conducted on protective measures toward mosquito bites at home (S6 Table). “Adopt at least 1 protective measure” (AOR: 4.05, 95% CI: 1.67–9.67), “adopt 3 or more protective measures” (AOR: 2.00, 95% CI: 1.25–3.20) and “adopt indoor mosquito protective measures” (AOR: 3.83, 95% CI:2.00–7.35) were positively associated with mosquito bites at home, with the adjustment of age, gender, living floor level and living near bushy, grassy area.

## Discussion

### Summary of findings

In this study, more than 92% of the respondents were aware of the announcement of local dengue fever outbreak in Hong Kong. For the knowledge of dengue fever, more than 90% understood that dengue is transmitted by mosquito. The symptoms that most of the respondents could identify were fever (84.0%), followed by headache (20.7%). About 70% of respondents have low risk perception of local dengue infection, though around 40% of the respondents reported that they were bitten by mosquitoes in August 2018. High risk locations included near grassy area, at respondent’s home and public transportation waiting spots. Near 80% of the respondents reported they had adopted at least one protective measure in the study period. Younger individuals (< 25 years old) and those who lived in lower floors (≤the 6th) and near bushy area were at higher risk of home mosquito bites. Regional ovitrap index was not significantly associated with the self-reported mosquito bites, but the adoption of mosquito protection was positively associated with self-reported bites at home.

### Mosquito bites pattern

Grassy areas are the favorable environment for mosquito breeding[32]. Consistent with this knowledge, the study result showed that people who live near these areas were more likely to get mosquito bites. To improve the condition, different stakeholders should collaborate for mosquito controls especially near grassy areas.

In terms of the demographic factors, younger age was associated with higher rate of self-reported mosquito bites, while the group aged 65 or above was less likely to report mosquito bites at home. However, literature suggested that people at older age were associated with weaker immediate as well as delayed mosquito bite reactions[33] by *Aedes albopictus*. Therefore, the lower biting rate in older adults reported might be related to their lower sensitivity of mosquito bites. Moreover, recall bias among the older groups might also contribute to the lower self-reported biting rate. Further studies exploring the biological mechanisms and other possible barriers of this observation will be needed.

### Association of mosquito bite and protective measure

Although protective measures were supposed to reduce the risk of mosquito bites, the relationship between them could not be found in this study. The positive association between mosquito bites and uptake of protective measures identified in this study was consistent with another cross-sectional study [34]. A possible explanation is that people who were affected by mosquito bites are more willing to apply protective measures due to their needs. Experimental study will be needed to assess the effectiveness of various protective measures.

For the bite pattern, it was found that outdoor environments were more prevalent for getting bite than at home, where locations near grassy area and at the transportation spot were the most common areas for getting bite. Given the high risk of getting bitten in public locations and the vector control measures implemented by the government, stronger mosquito prevention and control on public environments and more effective personal protective behaviors should be advocated.

### Uptake of protective measures

The most common adopted mosquito prevention measures were removing stagnant water at home, wearing long light color clothing, and using mosquito repellent. These measures were the main mosquito prevention measures promoted by Hong Kong government[35]. Such high prevalence of adoption might reflect that the public promotion had impacted on public’s preference in mosquito prevention. In comparing the measures adoption changes between before and after the local dengue cases, we found that removing stagnant water was significantly increased, but the use of electronic repellent and mosquito bracelet decreased. However, the reason for the decrease could not be concluded in this study.

#### Adopting indoor and outdoor measures

Furthermore, perceiving that dengue fever will have a high impact to society was associated with the adoption of protective measures (indoor, outdoor, at least 1 measure and more than 3 measures) in the analyses. Our results showed that residential factors (living in New Territories, near grassy area and construction area) were associated with the uptake of indoor protective measures. This finding was consistent with previous studies that rural area and construction area were reported to be at high risk of mosquito breeding [32,36]. As for the outdoor protective measures, other socio-demographic factors (younger age, female gender, higher education level) were the significant predictors. This may be related to their mosquito bite frequency, as younger age and female were more likely to get bitten as mentioned above. A study in France[12] also found that higher educational level and female gender were associated with the increased number of protective measures taken. However, a Malaysian study found no association between sociodemographic factors (including education level) and protective practices [37].

In addition, respondents who perceived greater impact of dengue fever on the society were associated with higher odds in adopting protective measures. More than half of the respondents who did not apply any protective measures because they did not regard protective measures as necessary. This was also consistent with previous studies that risk perception was one of the main determinants of behaviors in avoiding infection[38,39]. Awareness regarding dengue and the control of dengue risk should be reinforced in order to increase the uptake rate of protective measures.

### Knowledge of dengue

The most recognized symptom in this study was fever (84.0%), followed by headache (20.7%). The proportion of respondents knowing fever as a symptom of dengue was higher in comparison to other dengue-endemic regions, including India[40] (59.4%), Pakistan[41] (80.2%) and Jamaica[42] (49.5%), except for Costa Rica[43] (89%). However, for the other more distinctive symptoms like muscle and joint pain and pain behind the eyes, were less likely to be recognized. The difference may be associated with the local familiarity of dengue fever due to its prevalence.

The knowledge of dengue fever was not found to be associated with the uptake of mosquito prevention in this study. This result was in line with results of other studies from India[16], Singapore[44], Jamaica[42] and Pakistan[15], which suggested the presence of knowledge-practice gap. However, a high recognition of symptoms was found to be associated with better practices of mosquito protective measures in Costa Rica[43]. Although the association between knowledge of dengue and the willingness to adopt protective measures were uncertain, relevant knowledge should be beneficial to disease detection rate. Unable to recognize distinctive symptoms other than fever and headaches might increase barriers in detecting dengue infection, since both symptoms were commonly seen in respiratory conditions such as common cold and influenza. Insufficient education on the symptoms of dengue fever had been reported as one of the possible reasons for the low recognition of symptoms of dengue fever[14]. Knowledge about dengue’s symptoms should be increased to improve detection rate and possibly protections uptake rate.

### Relationship of mosquito bite and regional ovitrap index

Ovitrap is a small plastic container for detecting the larval breeding rate of *Aedes albopictus* mosquitos[45]. The ovitrap index for each district reflects the prevalence of *Aedes albopictus*. The index over 20% means that one-fifth of the surveyed ovitraps in that area have the eggs of *Aedes albopictus*, and additional pest control operations are suggested to conduct to eliminate mosquitos[30].

The regional ovitrap index in August was not found to be associated with the self-reported general mosquito bite at home. There were few possible reasons to explain this finding. Firstly, there were 5 other common mosquito types in Hong Kong besides the *Aedes*[20] but we do not have the information about the other mosquito types. Hence, in the analysis, a portion of mosquitos was not considered, which could also affect the self-reported mosquito bite. Secondly, the ovitrap index only measures the coverage of *Aedes albopictus* mosquito but not the density or total number of *Aedes albopictus* mosquito in the respective district[19]. Thirdly, the ovitraps used in the study were artificial containers added in their natural breeding ground. The breeding sites might compete with each other[46] and cause bias. Lastly, the ovitrap index used in our study only indicated the coverage of the *Aedes* in the whole district. It might not be directly associated to the situation of the residents. A review of the ovitrap placement location might be required.

#### Lion Rock Park dengue outbreak and the utility of ovitrap data

On 15 August 2018, the Hong Kong government announced the first local dengue outbreak within Hong Kong in the year[21]. The source of the outbreak was traced back to Lion Rock Park. The closest ovitrap location is in Wong Tai Sin Central. Although it did not reside in the top 10 high ovitrap index regions in July nor August among all 57 surveyed locations, the ovitrap index in that area remained high from May to July (23.4% to 21.8%). Other than Wong Tai Sin Central, only two more surveyed locations were up to that high level in this period. To our knowledge, although the ovitrap index did not directly reflect the possibility of outbreak[46], it provided information for identifying the environmental conditions that enhance the spread of dengue fever prior to the outbreak, which is useful for public health surveillance, as well as the designing the preparedness and response plan in the future. To further utilize the ovitrap index and fully reflect the situation of mosquito problem in that district, ovitraps could be placed at more locations and the locations should be chosen more carefully.

### Implication

This study found that the knowledge about the health impacts and the symptoms of the dengue fever among subjects were limited among Hong Kong population. Government should inform the public about the potential threat of the dengue and also help them to distinguish if they were infected easily. As for the individual/household preparedness, younger age, female gender, participants who living on lower level were reported to be the high-risk groups for getting mosquito bites at home. To further reinforce the protection, stronger prevention measures should be adopted by the government since mosquito bites were more commonly occurred in public places. At household and individual level, mosquito net could be considered due to its proved effectiveness[47] and the low usage rated found in the survey.

### Strengths and limitations

To the authors’ knowledge, this is the first study providing evidence in regard of mosquito bite patterns and protective measures adoptions in a non-dengue-endemic subtropical urban city. Apart from the report of mosquito bites and mosquito prevention measures, this study also associated subject’s residential environment and their perception with the uptake of the protective measures.

There were few limitations found in the study. Firstly, this study only assessed bite patterns in one month. The period prevalence of getting bitten in our study might not reflect the whole summer, although the study was conducted in one of the months with the highest number of ovitrap index recorded in a year. Secondly, the cross-sectional survey could not explicitly identify the temporal relationship between the mosquito protection measures, the mosquito bite status and the effectiveness of those measures. Nevertheless, the association patterns were consistent with a cross-sectional study about traveler and their mosquito prevention[34]. Thirdly, this study only assessed if respondents had applied protective measures but was not able to capture neither the frequency nor the quality of application due to the time constraint of telephone survey. Hence, further studies would need to include more related variables and investigate the effectiveness of the measures. Fourthly, it should also be highlighted that besides *Aedes albopictus*, there were another 5 common mosquitoes existing in Hong Kong[48]. Mosquito bite described in this study were not solely limited to dengue vectors but included other mosquito species. Hence, it might also explain the insignificant association of ovitrap index and mosquito bite. Fifthly, the self-reported approach adopted in this study might raise the issue of accuracy in estimating prevalence of mosquito bites and the use of protective measures, especially the recall ability of the subjects could be affected by age[49]. Lastly, this is a telephone survey study which households did not use a landline would not be reached.

## Conclusion

This study explored the knowledge of dengue fever, pattern of mosquito bites, use of protective measures and their associating factors among Hong Kong population using a cross-sectional random telephone survey 3 weeks after a dengue outbreak. Despite the high awareness of dengue outbreak, knowledge of symptoms and uptake rate of protective measures against mosquito bites in the subtropical city, mosquito bites were common in public places in summer months. This suggested the effectiveness of current protective measures was needed to be improved, especially for the grassy area and construction area near residents. Participants with high perception of risk to society were more likely to adopted protective measures against mosquito bites. Strategic anti-mosquito bite policies should target on raising the risk perception of local dengue, and target on males and people with lower education level.

## Supporting information

S1 Data Maps of ovitrap index and self-reported biting rate in August 2018. (XLSX)

S1 Table Unweighted and weighted results for Fig 2 and Fig 3. (PDF)

S2 Table Associated factors of mosquito bites at home (weighted analysis). (PDF)

S3 Table Uptake rate of protective measures (N = 590) (weighted analysis). (PDF)

S4 Table Associated factors of the use of indoor and outdoor protective measures against mosquito bites (weighted analysis). (PDF)

S5 Table Associated factors of the use of protective measures against mosquito bites (weighted analysis). (PDF)

S6 Table Multivariable logistic regression for mosquito bite at home. (PDF)

## References

[1] World Health Organization. Dengue and severe dengue [Internet]. 2018 [cited 2019 Oct 31]. Available from: https://www.who.int/news-room/fact-sheets/detail/dengue-and-severe-dengue.

[2] Brady OJ, Gething PW, Bhatt S, Messina JP, Brownstein JS, Hoen AG, et al Refining the Global Spatial Limits of Dengue Virus Transmission by Evidence-Based Consensus. PLoS Negl Trop Dis. 2012 8;6(8):e1760 10.1371/journal.pntd.0001760 22880140 PMC3413714

[3] Wang E, Ni H, Xu R, Barrett ADT, Watowich SJ, Gubler DJ, et al Evolutionary relationships of endemic/epidemic and sylvatic dengue viruses. J Virol. 2000;74(7):3227–34. 10.1128/jvi.74.7.3227-3234.2000 10708439 PMC111823

[4] Thai KTD, Nishiura H, Hoang PL, Tran NTT, Phan GT, Le HQ, et al Age-specificity of clinical dengue during primary and secondary infections. PLoS Negl Trop Dis. 2011 10.1371/journal.pntd.0001180 21713018 PMC3119638

[5] Karyanti MR, Uiterwaal CSPM, Kusriastuti R, Hadinegoro SR, Rovers MM, Heesterbeek H, et al The changing incidence of Dengue Haemorrhagic Fever in Indonesia: A 45-year registry-based analysis. BMC Infect Dis. 2014;14:412 10.1186/1471-2334-14-412 25064368 PMC4122763

[6] Hales S, De Wet N, Maindonald J, Woodward A. Potential effect of population and climate changes on global distribution of dengue fever: An empirical model. Lancet. 2002;360(9336):830–4. 10.1016/S0140-6736(02)09964-6 12243917

[7] Wilder-Smith A, Quam M, Sessions O, Rocklov J, Liu-Helmersson J, Franco L, et al The 2012 dengue outbreak in Madeira: Exploring the origins. Eurosurveillance. 2014;19(8):20718 10.2807/1560-7917.es2014.19.8.20718 24602277

[8] Lee JS, Farlow A. The threat of climate change to non-dengue-endemic countries: Increasing risk of dengue transmission potential using climate and non-climate datasets. BMC Public Health. 2019 10.1186/s12889-019-7282-3 31296193 PMC6625070

[9] Hetzel MW, Morris H, Tarongka N, Barnadas C, Pulford J, Makita L, et al Prevalence of malaria across Papua New Guinea after initial roll-out of insecticide-treated mosquito nets. Trop Med Int Heal. 2015;20(12):1745–55. 10.1111/tmi.12616 26427024

[10] Eyobo MB, Awur AC, Wani G, Julla AI, Remijo CD, Sebit B, et al Malaria indicator survey 2009, South Sudan: Baseline results at household level. Malar J. 2014;13:45 10.1186/1475-2875-13-45 24490895 PMC3922095

[11] Noland GS, Graves PM, Sallau A, Eigege A, Emukah E, Patterson AE, et al Malaria prevalence, anemia and baseline intervention coverage prior to mass net distributions in Abia and Plateau States, Nigeria. BMC Infect Dis. 2014;14:168 10.1186/1471-2334-14-168 24669881 PMC3994282

[12] Raude J, Chinfatt K, Huang P, Betansedi CO, Katumba K, Vernazza N, et al Public perceptions and behaviours related to the risk of infection with Aedes mosquito-borne diseases: A cross-sectional study in Southeastern France. BMJ Open. 2012;2(6). 10.1136/bmjopen-2012-002094 23194955 PMC3533069

[13] Trumbo CW, Harper R. Perceptual influences on self-protective behavior for West Nile virus, A survey in Colorado, USA Health behavior, health promotion and society. BMC Public Health. 2015;15:557 10.1186/s12889-015-1918-8 26082139 PMC4470069

[14] Dhimal M, Aryal KK, Dhimal ML, Gautam I, Singh SP, Bhusal CL, et al Knowledge, attitude and practice regarding dengue fever among the healthy population of highland and lowland communities in Central Nepal. PLoS One. 2014;9(7). 10.1371/journal.pone.0102028 25007284 PMC4090170

[15] Harapan H, Rajamoorthy Y, Anwar S, Bustamam A, Radiansyah A, Angraini P, et al Knowledge, attitude, and practice regarding dengue virus infection among inhabitants of Aceh, Indonesia: A cross-sectional study. BMC Infect Dis. 2018;18(1):96 10.1186/s12879-018-3006-z 29486714 PMC5830327

[16] Health Nagoor P. Knowledge, attitude and practice on dengue fever and its prevention and control measures in urban slums of South India. Int J Community Med. 2017;4(8).

[17] Pang J, Hildon ZJL, Thein TL, Jin J, Leo YS. Assessing changes in knowledge, attitude and practices on dengue diagnosis and management among primary care physicians after the largest dengue epidemic in Singapore. BMC Infect Dis. 2017;17(1):428 10.1186/s12879-017-2525-3 28619082 PMC5472871

[18] Centre for Health Protection. A Three-year Strategic Plan for the Prevention and Control of Dengue Fever in Hong Kong [Internet]. 2005. Available from: https://www.chp.gov.hk/files/pdf/scvbd_200508.pdf.

[19] Food and Environmental Hygiene Department of the Government of the Hong Kong Special Administrative Region. Vector-borne diseases: Dengue Fever [Internet]. 2018 [cited 2019 Oct 31]. Available from: https://www.fehd.gov.hk/english/pestcontrol/dengue_fever/index.html.

[20] Food and Environmental Hygiene Department of the Government of the Hong Kong Special Administrative Region. Public Services: Pest Control [Internet]. 2018 [cited 2019 Oct 31]. Available from: https://www.fehd.gov.hk/english/pestcontrol/Pcas.html.

[21] The Government of the Hong Kong Speical Administrative Region. Press Release: DH provides updates on follow-up on local cases of dengue fever [Internet]. [cited 2019 Oct 31]. Available from: https://www.info.gov.hk/gia/general/201808/15/P2018081500892.htm.

[22] The Government of the Hong Kong Speical Administrative Region. Press Release: Update on number of dengue fever cases [Internet]. [cited 2019 Oct 31]. Available from: https://www.info.gov.hk/gia/general/201811/23/P2018112300761.htm.

[23] Centre for Health Protection of the Department of Health of the Government of the Hong Kong Special Administrative Region. Number of notifiable infectious diseases by month in 2018 [Internet]. 2018 [cited 2019 Oct 31]. Available from: https://www.chp.gov.hk/en/statistics/data/10/26/43/6794.html.

[24] Benitez MA. Climate change could affect mosquito-borne diseases in Asia. Lancet. 2009;373(9669):P1070 10.1016/s0140-6736(09)60634-6 19338070

[25] Alto BW, Juliano SA. Precipitation and Temperature Effects on Populations of Aedes albopictus (Diptera: Culicidae): Implications for Range Expansion [Internet]. Vol. 38, Journal of Medical Entomology. 2001 [cited 2019 Oct 31]. p. 646–56. Available from: http://www.hko.gov.hk/climate_change/ClimProj20140317-e.pdf. 10.1603/0022-2585-38.5.646 11580037 PMC2579929

[26] Hong Kong Observatory. Projections of Hong Kong Climate for the 21st Century [Internet]. 2014. Available from: https://www.hko.gov.hk/en/press/files/ClimProj20140317-e.pdf

[27] The Government of the Hong Kong Speical Administrative Region. CHP investigates local cases of dengue fever [Internet]. [cited 2019 Oct 31]. Available from: https://www.ofca.gov.hk/en/data_statistics/data_statistics/key_stat/index.html.

[28] Office of the Communications Authority of the Government of the Hong Kong Special Administrative Region. Key Communication Statistics (Oct 2018) [Internet]. [cited 2018 Oct 31]. Available from: https://www.ofca.gov.hk/en/data_statistics/data_statistics/key_stat/.

[29] Chan EYY, Cheng CKY, Tam G, Huang Z, Lee P. Knowledge, attitudes, and practices of Hong Kong population towards human A/H7N9 influenza pandemic preparedness, China, 2014. BMC Public Health [Internet]. 2015;15:943 Available from: http://www.biomedcentral.com/1471-2458/15/943. 10.1186/s12889-015-2245-9 26395243 PMC4579795

[30] Food and Environmental Hygiene Department. Dengue fever: Dengue fever ovitrap index update [Internet]. 2019 [cited 2019 Oct 30]. Available from: https://www.fehd.gov.hk/english/pestcontrol/dengue_fever/ovitrap_index.html.

[31] Census and Statistics Department of the Government of the Hong Kong Special Administrative Region. Main Table (By-Census Results) [Internet]. [cited 2019 Oct 31]. Available from: https://www.bycensus2016.gov.hk/en/bc-mt.html.

[32] Mendenhall IH, Manuel M, Moorthy M, Lee TTM, Low DHW, Missé D, et al Peridomestic Aedes malayensis and Aedes albopictus are capable vectors of arboviruses in cities. PLoS Negl Trop Dis. 2017 10.1371/journal.pntd.0005667 28650959 PMC5501678

[33] Oka K, Ohtaki N. Clinical observations of mosquito bite reactions in man: A survey of the relationship between age and bite reaction. J Dermatol. 1989;16(3):212–9. 10.1111/j.1346-8138.1989.tb01251.x 2571626

[34] Goodyer L, Song J. Mosquito bite-avoidance attitudes and behaviors in travelers at risk of malaria. J Travel Med. 2014;21(2):33–8. 10.1111/jtm.12053 24383652

[35] Centre for Health Protection (Department of Health of the Government of Hong Kong SAR). General measures on preventing mosquito bites and mosquito breeding [Internet]. 2018 [cited 2019 Nov 19]. Available from: https://www.chp.gov.hk/en/features/46196.html.

[36] Liang S, Hapuarachchi HC, Rajarethinam J, Koo C, Tang CS, Chong CS, et al Construction sites as an important driver of dengue transmission: Implications for disease control. BMC Infect Dis. 2018 10.1186/s12879-018-3311-6 30089479 PMC6083507

[37] Wong LP, Shakir SMM, Atefi N, AbuBakar S. Factors affecting dengue prevention practices: Nationwide survey of the Malaysian public. PLoS One. 2015;10(4):e0122890 10.1371/journal.pone.0122890 25836366 PMC4383514

[38] Brewer NT, Chapman GB, Gibbons FX, Gerrard M, McCaul KD, Weinstein ND. Meta-analysis of the relationship between risk perception and health behavior: The example of vaccination. Heal Psychol. 2007;26(2):136–45. 10.1037/0278-6133.26.2.136 17385964

[39] Shadick NA, Daltroy LH, Phillips CB, Liang US, Liang MH. Determinants of tick-avoidance behaviors in an endemic area for Lyme disease. Am J Prev Med. 1997;13(4):365–70. 9236962

[40] Jeelani S, Sabesan S, Subramanian S. Community knowledge, awareness and preventive practices regarding dengue fever in Puducherry—South India. Public Health. 2015;129(6):790–6. 10.1016/j.puhe.2015.02.026 25863688

[41] Ramzan M, Ansar A, Nadeem S. Dengue epidemics: Knowledge perhaps is the only key to success. J Ayub Med Coll Abbottabad. 2015;27(2):402–6. 26411128

[42] Shuaib F, Todd D, Campbell-Stennett D, Ehiri J, Jolly PE. Knowledge, attitudes and practices regarding dengue infection in Westmoreland, Jamaica. West Indian Med J. 2010;59(2):139–46. 21132094 PMC2996104

[43] Egedus VL, Morales OJ, Alfaro OA. Knowledge, perceptions, and practices with respect to the prevention of dengue in a mid-Pacific coastal village of Costa Rica. Rev Biol Trop. 2014;62(3):859–67. 10.15517/rbt.v62i3.14065 25412518

[44] Ong DQR, Sitaram N, Rajakulendran M, Koh GCH, Seow ALH, Ong ESL, et al Knowledge and practice of household mosquito breeding control measures between a dengue hotspot and non-hotspot in Singapore. Ann Acad Med Singapore. 2010;39(2):146–9. 20237738

[45] Centre for Health Protection. Scientific committee on vector-borne diseases update on epidemiology, prevention and control of dengue fever in Hong Kong [Internet]. 2016. Available from: https://www.hko.gov.hk/en/press/files/ClimProj20140317-e.pdf.

[46] Sivagnaname N, Gunasekaran K. Need for an efficient adult trap for the surveillance of dengue vectors. Indian Journal of Medical Research. 2012 23287120 PMC3573594

[47] Van Remoortel H, De Buck E, Singhal M, Vandekerckhove P, Agarwal SP. Effectiveness of insecticide-treated and untreated nets to prevent malaria in India. Tropical Medicine and International Health. 2015 10.1111/tmi.12522 25877758

[48] Food and Environmental Hygiene Department of the Government of the Hong Kong Special Administrative Region. Pest Control: Mosquito [Internet]. 2018 [cited 2019 Oct 31]. Available from: https://www.fehd.gov.hk/english/pestcontrol/risk-pest-mosquito.html.

[49] Stephan Y, Sutin AR, Caudroit J, Terracciano A. Subjective age and changes in memory in older adults. Journals Gerontol—Ser B Psychol Sci Soc Sci. 2016;71(4):675–83. 10.1093/geronb/gbv010 25748213

