# Supplementary material for: Sociodemographic predictors of knowledge, mosquito bite patterns and protective behaviors concerning vector borne disease: The case of dengue fever in Chinese subtropical city, Hong Kong
Source: PLoS Negl Trop Dis. 2021 Jan 19;15(1):e0008993. doi: 10.1371/journal.pntd.0008993 (PMC7846016; doi:10.1371/journal.pntd.0008993)
Supplement: S1 Table — (PDF) [file pntd.0008993.s002.pdf]

S1 Table. Unweighted and weighted results for Fig 2 and Fig 3

Fig. 2 Symptoms of dengue fever recognized by respondents in September 2018		
	Unweighted	Weighted
Fever	83.9%	83.1%
Headache	20.7%	20.3%
Vomiting	12.4%	13.3%
Muscle and joint pain	11.9%	12.2%
Rash	4.4%	3.9%
Pain behind the eyes	3.1%	3.3%
Nausea	2.0%	1.6%
Swollen lymph node	0.7%	0.5%
Fig 3. The locations of mosquito bites in August 2018		
	Unweighted	Weighted
At home	42.6%	43.1%
Away from home	82.6%	84.1%
Near grass area	63.4%	64.2%
At transport waiting spot	39.6%	40.8%
At work	27.2%	29.6%
Near water source	19.1%	22.0%
Near construction areas	12.3%	12.2%
During transportation	11.9%	13.3%
