# Supplementary material for: Sociodemographic predictors of knowledge, mosquito bite patterns and protective behaviors concerning vector borne disease: The case of dengue fever in Chinese subtropical city, Hong Kong
Source: PLoS Negl Trop Dis. 2021 Jan 19;15(1):e0008993. doi: 10.1371/journal.pntd.0008993 (PMC7846016; doi:10.1371/journal.pntd.0008993)
Supplement: S2 Table — (PDF) [file pntd.0008993.s003.pdf]

S2 Table. Associated factors of mosquito bites at home (weighted analysis)

Factors	No mosquito bites (n=460)	Have mosquito bites (n=105)	p-value#	AOR (95% CI)	p-value$
Age			<0.001		
18-24	48 (10.4%)	19 (17.9%)		Ref.	
25-44	138 (30.0%)	49 (46.2%)		0.682 (0.349 – 1.332)	0.262
45-64	177 (38.5%)	30 (28.3%)		0.359 (0.170 – 0.757)	0.007*
65 or older	97 (21.1%)	8 (7.5%)		0.224 (0.076 – 0.664)	0.007*
Gender			0.087		
Male	186 (40.4%)	33 (31.4%)		Ref.	
Female	274 (59.6%)	72 (68.6%)		1.779 (1.0770 – 2.936)	0.024*
Residential district			0.844		
Hong Kong Island	76 (16.6%)	16 (15.2%)			
Kowloon	136 (29.6%)	34 (32.4%)			
New Territories	247 (53.8%)	55 (52.4%)			
Housing			0.754		
Public housing	189 (41.3%)	38 (36.2%)			
Support housing	68 (14.8%)	19 (18.1%)			
Private housing	197 (42.9%)	47 (44.8%)			
Temporary housing	4 (0.9%)	1 (1.0%)			
Floor Level			<0.001		
<6	85 (18.6%)	37 (35.6%)		Ref.	
6-25	277 (60.7%)	57 (54.8%)		0.378 (0.224 – 0.637)	<0.001*
>25	94 (20.6%)	10 (9.6%)		0.219 (0.100 – 0.480)	<0.001*
Live near water source			0.301		
No	269 (58.9%)	56 (53.3%)			
Yes	188 (41.1%)	49 (46.7%)			
Live near bushy and grassy area			0.019		
No	4 (10.0%)_	3 (2.9%)		Ref.	
Yes	413 (90.0%)	102 (97.1%)		4.036 (1.169 – 13.934)	0.027*
Live near construction site			0.405		
No	299 (65.9%)	64 (61.5%)			
Yes	155 (34.1%)	40 (38.5%)			
CSSA (Fisher)			0.050		
No	433 (94.5%)	102 (99.0%)		Ref.	
Yes	25 (5.5%)	1 (1.0%)		0.190 (0.291 – 1.846)	0.190
Education			0.010		
primary and below	120 (26.1%)	13 (12.3%)		Ref.	
secondary	199 (43.3%)	53 (50.0%)		1.379 (0.613 – 3.103)	0.437
post-secondary	141 (30.7%)	40 (37.7%)		1.226 (0.502 – 2.993)	0.655
Chronic disease			0.037		
No	344 (75.7%)	87 (85.3)		Ref.	
Yes	110 (24.2%)	15 (14.7%)		0.864 (0.435 – 1.714)	0.676
Regional ovitrap index	6.47 (4.97 – 11.05)	6.17 (4.97 – 11.05)	0.356		
