# Supplementary material for: Sociodemographic predictors of knowledge, mosquito bite patterns and protective behaviors concerning vector borne disease: The case of dengue fever in Chinese subtropical city, Hong Kong
Source: PLoS Negl Trop Dis. 2021 Jan 19;15(1):e0008993. doi: 10.1371/journal.pntd.0008993 (PMC7846016; doi:10.1371/journal.pntd.0008993)
Supplement: S3 Table — (PDF) [file pntd.0008993.s004.pdf]

S3 Table. Uptake rate of protective measures (N=590) (weighted analysis)

Mosquito protective measures	Before the local outbreak		After the local outbreak		McNemar's test
	N	%	N	%	p
**Indoor mosquito measures**	394	66.8%	400	67.8%	0.345
Removing stagnant water	300	51.0%	327	55.6%	<0.001
Electronic repellent	116	19.7%	89	14.6%	<0.001
Insecticide	111	18.8%	105	17.8%	0.238
Mosquito net on door and window	75	12.7%	73	12.4%	0.625
Mosquito incense	51	8.7%	48	8.1%	0.549
Mosquito plant	16	2.7%	14	2.4	0.500
Mosquito net during sleep	15	2.5%	15	2.5%	1.000
Detergent cleaning	14	2.4%	15	2.5%	1.000
**Outdoor mosquito measures**	314	53.2%	300	50.8%	0.049
Mosquito chemical repellent	234	39.9%	225	38.4%	0.243
Light colored long clothing	166	28.1%	173	29.3%	0.230
Mosquito sticker	110	18.6%	103	17.5%	0.265
Mosquito bracelet	38	6.4%	24	4.1%	0.00
