# Supplementary material for: Sociodemographic predictors of knowledge, mosquito bite patterns and protective behaviors concerning vector borne disease: The case of dengue fever in Chinese subtropical city, Hong Kong
Source: PLoS Negl Trop Dis. 2021 Jan 19;15(1):e0008993. doi: 10.1371/journal.pntd.0008993 (PMC7846016; doi:10.1371/journal.pntd.0008993)
Supplement: S4 Table — (PDF) [file pntd.0008993.s005.pdf]

S4 Table. Associated factors of the use of indoor and outdoor protective measures against mosquito bites (weighted analysis)

	Adopt indoor measure		Adopt outdoor measure	
	AOR (95% CI)	p	AOR (95% CI)	p
Age				
18-24	Ref.		Ref.	
25-44	0.931 (0.485 – 1.786)	0.830	0.854 (0.438 – 1.666)	0.643
45-64	1.138 (0.591 – 2.190)	0.706	0.484 (0.246 – 0.950)	0.035*
65 or older	1.149 (0.558 – 2.369)	0.714	0.280 (0.124 – 0.629)	0.002*
Gender				
Male	Ref.		Ref.	
Female	1.165 (0.783 – 1.735)	0.457	2.055 (1.361 – 3.102)	0.001*
Residential district				
Hong Kong Island	Ref.		Ref.	
Kowloon	1.950 (1.109 – 3.428)	0.022*	1.364 (0.766 – 2.428)	
New Territories	1.946 (1.160 – 3.264)	0.013*	1.872 (1.105 – 3.171)	0.020*
Floor level				
<6	Ref.			
6-25	0.422 (0.248 – 0.717)	0.002*		
>25	0.429 (0.228 – 0.808)	0.010*		
Live near water source				
No			Ref.	
Yes			1.247 (0.826 – 1.881)	0.294
Live near bushy, grass area				
No	Ref.		Ref.	
Yes	2.542 (1.331 – 4.852)	0.005*	1.328 (0.662 – 2.662)	0.424
Live near construction site				
No	Ref.		Ref.	
Yes	1.692 (1.118 – 2.562)	0.014*	1.668 (1.113 – 2.501)	0.013*
Education				
Primary and below			Ref.	
Secondary			2.327 (1.310 – 4.135)	0.004*
Post-secondary			2.751 (1.423 – 5.318)	0.003*
Perceived mosquito bites affecting their daily life (Mosquito Annoyance)				
No	Ref.		Ref.	
Yes	1.826 (0.850 – 3.922)	0.129	0.866 (0.428 – 1.750)	0.688
Dengue fever could be avoided through individual/household prevention				
Disagree/Neutral	Ref.		Ref.	
Agree	1.695 (1.141 – 2.519)	0.010*	1.167 (0.785 – 1.736)	0.445
The impact of dengue toward the whole society				
Low	Ref.		Ref.	
Medium	1.464 (0.956 – 2.242)	0.084	2.244 (1.454 – 3.464)	<0.001*
High	2.029 (1.200 – 3.430)	0.009*	3.200 (1.901 – 5.387)	<0.001*
Risk for getting dengue fever in Hong Kong				
Very low/low			Ref.	
Medium/ very high			1.295 (0.821 – 2.044)	0.267

*p<0.05
