# Supplementary material for: Sociodemographic predictors of knowledge, mosquito bite patterns and protective behaviors concerning vector borne disease: The case of dengue fever in Chinese subtropical city, Hong Kong
Source: PLoS Negl Trop Dis. 2021 Jan 19;15(1):e0008993. doi: 10.1371/journal.pntd.0008993 (PMC7846016; doi:10.1371/journal.pntd.0008993)
Supplement: S5 Table — (PDF) [file pntd.0008993.s006.pdf]

S5 Table. Associated factors of the use of protective measures against mosquito bites (weighted analysis)

	Adopt at least 1 protective measure		Adopt 3 or more protective measures	
	AOR (95% CI)	p	AOR (95% CI)	p
Age				
18-24	Ref		Ref.	
25-44	0.784 (0.324 – 1.898)	0.590	0.833 (0.437 – 1.587)	0.579
45-64	0.498 (0.208 – 1.193)	0.118	0.519 (0.264 – 1.047)	0.061
65 or older	0.443 (0.166 – 1.180)	0.103	0.437 (0.182 – 1.047)	0.063
Gender				
Male	Ref		Ref.	
Female	1.552 (0.964 – 2.500)	0.070	1.680 (1.085 – 2.601)	0.020*
Residential district				
Hong Kong Island	Ref		Ref.	
Kowloon	2.139 (1.116 – 4.101)	0.022*	0.984 (0.503 – 1.924)	0.963
New Territories	1.570 (0.883 – 2.791)	0.125	2.502 (1.370 – 4.572)	0.003*
Floor level				
<6	Ref		Ref.	
6-25	0.410 (0.215 – 0.780)	0.007*	0.333 (0.201 – 0.551)	<0.001*
>25	0.591 (0.272 – 1.287)	0.186	0.282 (0.149 – 0.534)	<0.001*
Live near water source				
No	Ref		Ref.	
Yes	1.148 (0.701 – 1.877)	0.582	1.208 (0.788 – 1.852)	0.386
Live near bushy, grass area				
No	Ref		Ref.	
Yes	2.603 (1.269 – 5.337)	0.009*	1.889 (0.808 – 4.418)	0.142
Live near construction site				
No	Ref		Ref.	
Yes	2.009 (1.191 – 3.387)	0.009*	1.923 (1.264 – 2.926)	0.002*
Education				
Primary and below	Ref		Ref.	
Secondary	1.197 (0.644 – 2.223)	0.570	3.839 (1.899 – 7.759)	<0.001*
Post-secondary	0.939 (0.457 – 1.926)	0.863	2.740 (1.268 – 5.918)	0.010*
Perceived mosquito bites affecting their daily life (Mosquito Annoyance)				
No	Ref		Ref.	
Yes	2.103 (0.697 – 6.344)	0.187	1.641 (0.823 – 3.273)	0.159
Dengue fever could be prevented through individual/household protection				
Disagree/Neutral	Ref		Ref.	
Agree	1.160 (0.730 – 1.845)	0.529	2.011 (1.287 – 3.142)	0.002
The impact of dengue toward the whole society				
Low	Ref		Ref.	
Medium	2.109 (1.273 – 3.494)	0.004*	1.393 (0.860 – 2.256)	0.178
High	2.797 (1.465 – 5.341)	0.002*	2.403 (1.393 – 4.145)	0.002*
Risk for getting dengue fever in Hong Kong				
Very low/low			Ref.	
Medium/ very high			1.001 (0.617 – 1.623)	0.998
