# Supplementary material for: Sociodemographic predictors of knowledge, mosquito bite patterns and protective behaviors concerning vector borne disease: The case of dengue fever in Chinese subtropical city, Hong Kong
Source: PLoS Negl Trop Dis. 2021 Jan 19;15(1):e0008993. doi: 10.1371/journal.pntd.0008993 (PMC7846016; doi:10.1371/journal.pntd.0008993)
Supplement: S6 Table — (PDF) [file pntd.0008993.s007.pdf]

S6 Table. Multivariable logistic regression for mosquito bite at home

Factor	Unweighted	Weighted
	AOR (95% CI)	AOR (95% CI)
Adopt at least 1 protective measure	4.05 (1.67 – 9.67) $p<0.05$	2.99 (1.34 – 6.67) $p=0.008$
Adopt 3 or more protective measures	2.00 (1.25 – 3.20) $p<0.05$	1.50 (0.94 – 2.40) $p=0.086$
Adopt indoor mosquito protective measures	3.83 (2.00 – 7.35) $p<0.05$	3.62 (1.90 – 6.90) $p<0.001$
